# Perceptions of COVID-19 Vaccine, Racism, and Social Vulnerability: An Examination among East Asian Americans, Southeast Asian Americans, South Asian Americans, and Others

**DOI:** 10.3390/vaccines10081333

**Published:** 2022-08-17

**Authors:** Tsu-Yin Wu, Olivia Ford, Alice Jo Rainville, Xining Yang, Chong Man Chow, Sarah Lally, Rachel Bessire, Jessica Donnelly

**Affiliations:** 1 Center for Health Disparities Innovations and Studies, Eastern Michigan University, Ypsilanti, MI 48197, USA; 2 Dietetics and Human Nutrition, Eastern Michigan University, Ypsilanti, MI 48197, USA; 3 Human Nutrition, Eastern Michigan University, Ypsilanti, MI 48197, USA; 4 Geography and Geology, Eastern Michigan University, Ypsilanti, MI 48197, USA; 5 Psychology, Eastern Michigan University, Ypsilanti, MI 48197, USA

**Keywords:** COVID-19 vaccines, Asian Americans, COVID-19, social vulnerability, racism

## Abstract

As COVID-19 vaccines are readily available and most U.S. adults who are enthusiastic about the vaccine have received it, motivating those who have not been vaccinated to accept it has become a challenge. The purpose of this study was to understand the mechanisms behind COVID-19 vaccine acceptance in Asian American ethnic groups, including how sociodemographic characteristics and racism predict COVID-19 and vaccine perceptions. The study also examined associations between social vulnerability and COVID-19 and vaccine perceptions. Social vulnerability is defined as the degree to which a community is able to prepare and respond to a natural or man-made disaster. This cross-sectional study used community-based survey data collected from April to September 2021. Study measures included demographics, perceptions of COVID-19 and COVID-19 vaccines, and racism-related experiences. The results showed that, compared to Non-Asians, East Asians reported that they had significantly more challenges accessing COVID-19 vaccines, and South Asians reported significantly higher safety concerns about COVID-19 vaccines. Our study also found that racism experience mediates the association between race/ethnicity and safety concerns about COVID-19 vaccines. Three Asian subgroups (East Asians, South Asians, and Southeast Asians) experienced more racism (compared to Non-Asians), and more experience of racism was related to greater safety concerns. Geographical Information System (GIS) maps revealed that residents of lower social vulnerability index (SVI) areas reported fewer unfairness perceptions and that higher SVI areas had lower vaccine accessibility and trust in public health agencies. Our study advances the understanding of racism, social vulnerability, and COVID-19 vaccine-related perceptions among Asian Americans. The findings have implications for policymakers and community leaders with respect to tailoring COVID-19 program efforts for socially vulnerable populations and Asian American groups that experience greater challenges regarding vaccine safety concerns and accessibility.

## 1. Introduction

Although COVID-19 vaccines have been readily available in the United States (U.S.) for over a year as of June 2022, the rate of vaccination has slowed [1]. In July 2022, the Centers for Disease Control and Prevention (CDC) reported that 74% of people had received at least one dose of the vaccine and about 40% of the fully vaccinated population had also received a booster vaccine [2]. Having reached the point where most adults living in the U.S. who were enthusiastic about the vaccine have received it, the challenge is motivating those who have not been vaccinated to accept the vaccine. An in-depth understanding of perceptions of COVID-19 and COVID-19 vaccines can assist in effective planning for tailored efforts to increase vaccine uptake. This study aimed to assess such perceptions, specifically vaccine concerns among Asian Americans living in Michigan. Vaccine concerns are defined as the collective patterns of attitudes towards and beliefs about a vaccine and can reflect the meanings and reasonings individuals attribute to their vaccination decisions [3,4].

As racial and ethnic minorities have been disproportionately affected by COVID-19 in terms of both severity of infection and mortality rates [5], racial and ethnic disparities in vaccination rates are of increasing concern. Asian Americans comprise 6% of the total population of the United States and are the fastest-growing ethnic group [6,7]. They have dealt with many societal pressures during the COVID-19 pandemic that could result in barriers to vaccination, including language, anti-Asian racial discrimination, and medical and institutional distrust, among others [7,8,9,10].

Wagner and colleagues [11] conducted a survey among Detroit residents to assess personal experiences of COVID-19 and how trust in authorities mediates racial disparities in vaccination acceptance. Participants represented several racial/ethnic groups, including Non-Hispanic Black, Non-Hispanic White, Hispanic, and Asian American, Pacific Islander/Native American groups. The results showed that trust in healthcare providers significantly differed across racial/ethnic groups, with Non-Hispanic Whites reporting the greatest level. Trust in the government was not significantly different across the racial/ethnic groups. The factor that was associated with vaccine intent across racial groups was a recommendation from a healthcare provider; however, Non-Hispanic Blacks and Asian Americans were least likely to be influenced. That having been said, lack of diversity in COVID-19 vaccine trials and potential differences in vaccine efficacy with respect to race [12] may also be factors in the hesitancy of people of ethnic backgrounds to accept COVID-19 vaccines as being safe for them [13].

In terms of sources of COVID-19 vaccine information, Latkin and colleagues [14] conducted a four-wave longitudinal study using online surveys and found that respondents who reported greater trust in information from the CDC, state health departments, mainstream news, and a well-known university (Johns Hopkins University) were also more likely to trust COVID-19 vaccines. In addition, men were more likely to trust COVID-19 vaccines than women.

Park and colleagues [15] conducted a national survey of Asian Americans and Pacific Islanders and found that Vietnamese respondents were more willing to be vaccinated (57.2%) than Chinese (40.3%), Filipinos (38%), and Koreans (33.8%). In another U.S.-based study with racial/ethnic groups conducted in early 2021 [16], researchers found that Asian Americans reported the highest intention to be vaccinated among all respondents, with 39.2% of Asian Americans reporting that they were extremely likely to be vaccinated. Among those, three subgroups, Asian Indian (46.6%), Filipino (39.7%), and other Asian (37.7%), reported that they were extremely likely to be vaccinated. However, a greater percentage of Asian American respondents (11.5%) also reported having concerns about where to access the vaccine compared to overall respondents (10.3%).

Despite Asian Americans generally being less vaccine-hesitant compared to other minority groups [17], it is important to examine factors that may affect COVID-19 vaccine perceptions given the unprecedented effects of the COVID-19 pandemic on this group, including anti-Asian American violence and racism. Perceptions, including concerns and worries, will provide valuable information that has yet to be fully researched [18] for the successful implementation of vaccine uptake by various Asian American ethnic groups. Many studies have not specifically focused on Asian Americans’ attitudes and beliefs about COVID-19 vaccines and, when surveyed, many Asian Americans have been unable to complete surveys and questionnaires due to limited English proficiency [19].

While a limited number of studies conducted with Asian Americans have examined their COVID-19 vaccination behaviors, to our knowledge, none has studied the mechanisms behind vaccine hesitancy and how racism and social vulnerability have affected vaccine perceptions. Socially vulnerable populations are at an increased risk of being marginalized and underserved during public health crises due to factors such as socioeconomic status, housing, transportation access, and being non-White [20]. The CDC Agency for Toxic Substances and Disease Registry (ASTDR) uses U.S. census variables to calculate social vulnerability index scores for census tracts [21] and these help identify communities in need of support during times of public health crises and disasters, such as the COVID-19 pandemic. The purpose of the current study was to obtain a comprehensive understanding of COVID-19 vaccine acceptance in Asian American ethnic groups. The study objectives included: (1) to compare perceptions (safety, concerns, accessibility, trust) of COVID-19 vaccinations among East, Southeast, South Asians, and Non-Asians; (2) to investigate how sociodemographic characteristics and racism are associated with COVID-19 perceptions; (3) to determine whether racism-related variables (unfairness and concerns about safety) mediate the effects of socio-demographics on COVID-19 vaccine perceptions; and (4) to examine the extent to which these perceptions are associated with social vulnerability using geographic information system (GIS)-generated maps. The findings from this study present a unique perspective on disparities faced by Asian Americans and provide insights into improving COVID-19 vaccination uptake in this public health crisis.

## 2. Materials and Methods

### 2.1. Study Design and Setting

A cross-sectional community-based survey was carried out in Michigan from April to September 2021. Michigan is home to the second-largest Asian American population in the Midwest. From 2014 to 2018, the percentage increase of Asian Americans in the state was approximately 17.9% [22]. According to the 2019 American Community Survey, 327,230 Asian Americans resided in Michigan, accounting for 3.3% of the population [23]. The Asian American population in Michigan includes different subgroups, with Asian Indian, Chinese, Filipino, Japanese, Korean, and Vietnamese subgroups accounting for approximately 75.3% of the Asian population [24]. 

### 2.2. Participants and Procedures

The research study was facilitated via a Center in Michigan, funded through a CDC Racial Ethnic Approaches to Community Health (REACH) cooperative agreement. The Center is one of 40 REACH recipients in the U.S. and the only recipient whose sole priority population is Asian Americans. Study eligibility criteria were self-identification as Asian American or Non-Asian, ability to read English or one of seven Asian languages (Chinese, Korean, Bangla, Thai, Tamil, Urdu, Vietnamese), and being 18 years of age or older. Respondents were recruited through Asian community organizations who serve Asian Americans, social media, ethnic newspapers, and personal networks. The surveys were administered at locations where Asian Americans gathered, including community centers, mosques, temples, churches, and nail salons, through a vaccine advocacy program serving Asian Americans in Michigan. A team of research staff was trained for data collection. Respondents provided informed consent prior to completing the survey, which took approximately 15 min. Participation was voluntary. The research study protocol was approved by the Eastern Michigan University Institutional Review Board (protocol no.: UHSRC-FY20-21-199; approved 25 March 2021). 

### 2.3. Measures

The study instrument was developed based on the framework provided by the Centers for Disease Control and Prevention COVID-19 Rapid Community Assessment [25] and included three sections. The first section assessed demographic variables, including Asian ethnic groups, which were later categorized into South Asian (Asian Indian, Bangladeshi, and Burmese), East Asian (Chinese, Japanese, and Taiwanese), Southeast Asian (Filipino, Korean, and Vietnamese), and Non-Asian (Arab, African America, Czech, Indian, Jewish, and White), sex, nativity (country of birth), years lived in the U.S., marital status, employment, education, household income, and self-rated level of English proficiency. The second section measured perceptions of COVID-19 and COVID-19 vaccines with the following study variables: (a) general concerns about getting COVID-19; (b) COVID-19 vaccine accessibility, measured by assessment of specific challenges related to receiving COVID-19 vaccines and general perceptions of difficulty accessing vaccines; (c) COVID-19 vaccine concern, measured using nine specific concerns and general perceptions of vaccine safety; and (d) trust in public health agencies. The last section of the survey measured respondents’ racism-related experience using two items: unfair treatment and safety concerns due to race/ethnicity. Racism was assessed by inquiring about participants’ perceptions of unfair treatment and worry about safety; these items were adopted from the Urban Institute’s work on unfair judgment and discrimination in healthcare settings [26]. Items measuring perceptions and racism used a six-point Likert scale. 

Data were collected in person and the surveys were made available in English and seven Asian languages (listed in Section 2.2, Participants and Procedures) using the standard back-translation technique [27].

### 2.4. Analyses

**Figure 1:**
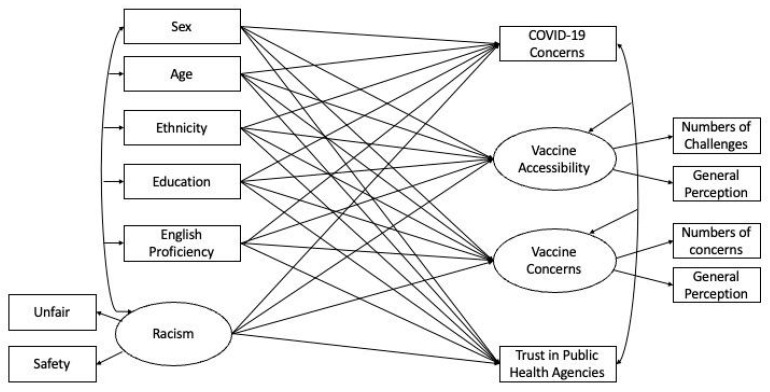
Structural equation model (SEM) predicting general COVID-19 concerns, COVID-19 vaccine accessibility, COVID-19 vaccine concerns, and trust in public health agencies using demographic characteristics and racism experience. The shared variance (*r*^2^) between indicators formed the latent variable: vaccine accessibility = 0.08; vaccine concerns = 0.14; and racism = 0.30. These statistics indicate the association and hence internal consistency among the items [30].

Data were analyzed using R version 4.0.3 [28]. Data analyses proceeded in four steps: (1) using descriptive statistics to summarize sample characteristics and study variables; (2) examining racial/ethnic group differences in relation to respondents’ racism experiences; and (3) predicting general COVID-19 concerns, COVID-19 vaccine accessibility, COVID-19 vaccine concerns, and trust in public health agencies using demographic characteristics and racism experience with a structural equation model (SEM) (see Figure 1). In this model, three latent variables (i.e., COVID-19 vaccine concerns, vaccine accessibility, and racism experience) were specified. The predictors as well as the outcome variables were allowed to covary (as represented by double-headed arrows). In addition, structural coefficients were used to represent the unique association of each predictor with the outcome variables (represented by single-headed arrows). The model fit of the SEM was examined based on a collection of indices, including CFI (>0.95), TLI (>0.95), and RMSEA (<0.05) [29]. Following these steps, (4) the mediation hypothesis which predicts that racism experience mediates the association between race/ethnicity and the outcome variables (general COVID-19 concerns, COVID-19 vaccine accessibility, COVID-19 vaccine concerns, and trust in public health agencies) would be tested. It is important to note that a mediation test was warranted only if racism experience was predictive of one or more of the outcome variables, above and beyond race/ethnicity. Thus, mediation paths were examined based on the results concluded by the SEM in the third step. 

For the objective of examining social vulnerability and COVID-19 vaccine concerns, we conducted a spatial analysis to assess the relationship between Zip Code Tabulation Area level Social Vulnerability Indices and COVID-19 confidence variables among survey respondents. The CDC Social Vulnerability Index (SVI) for the state of Michigan measures relative vulnerability within census tracts [31]. Using a crosswalk file [32], we aggregated the tract level SVI to zip code level and computed the weighted mean SVI using the overall population obtained from the U.S. Census 2019 American Community Survey 5-year estimate [33]. We used the zip codes reported by the survey respondents to pinpoint each record to its zip code area. In total, 590 records have been geocoded and the locations of survey respondents have been visualized using proportional symbols in the geographic information system (GIS) software ESRI ArcGIS Pro 2.8 [34]. 

## 3. Results

### 3.1. Descriptive Statistics

As seen in Table 1, among 617 respondents, the majority identified as female (58.82%), had a bachelor’s degree or higher (53.42%), and were employed (69.70%). In terms of ethnic breakdown, more respondents were East Asians (42.76%), followed by Southeast Asians (29.44%), South Asians (16.45%), and Non-Asians (11.35%). The respondents’ ages ranged from 18 to 80 years old (M = 40.91; SD = 14.23), and the mean English proficiency self-rating was 3.23 (SD = 0.83), with 4 representing a rating of very high. The descriptive statistics for COVID-19 and COVID-19 vaccine concerns, accessibility, and trust in public health agencies are presented in Table 1. 

### 3.2. Racial/Ethnic Differences Regarding Racism 

One-way ANOVAs (see Table 2) were conducted to compare racism experience variables (unfairness and safety) among groups, and the results show that all three Asian American groups reported that they were treated unfairly due to their race/ethnicity compared to the Non-Asian group. No significant difference was found within the three Asian groups. A similar pattern was found for worrying about safety related to one’s race/ethnicity since the onset of the pandemic. Each Asian American group reported significantly more worry about safety related to one’s race/ethnicity compared to the Non-Asian group, with East Asians reporting significantly more concern than South or Southeast Asians. There were no significant differences between the South and Southeast Asian groups with respect to safety concerns (Table 2).

### 3.3. Predicting COVID-19-Related Concerns with Mediation Effects

An SEM analysis was used to examine the relative importance of demographic characteristics and racism experience in predicting four perception outcomes: (a) general COVID-19 concerns, (b) COVID-19 vaccine accessibility, (c) COVID-19 vaccine concerns, and (d) trust in public health agencies (see Figure 1). The model fit for the SEM was excellent, with χ^2^ (153) = 76.23, CFI = 0.99, TLI = 0.95, and RMSEA = 0.04 [29]. The results of the SEM, specifically the regression weights of the predictors, are presented in Table 3.

When predicting general concern about getting COVID-19, males (compared to females) and older participants reported significantly lower levels of concerns. Compared to Non-Asians, East Asians had significantly lower concerns about getting COVID-19, while South and Southeast Asians were not significantly different from Non-Asians. Education had some effect on individuals’ general concerns about getting COVID-19, with those who graduated from high school or who earned a GED scoring significantly lower compared to those with less than a high school education. Employment status and racism were both found not to be significant in predicting general concern about getting COVID-19. However, higher English proficiency was predictive of higher general concern about getting COVID-19 (Table 3).

When predicting COVID-19 vaccine accessibility, East Asians (compared to Non-Asians) reported significantly more challenges when it came to COVID-19 vaccine accessibility, while South and Southeast Asians were not significantly different compared to Non-Asians. Demographic variables (sex, education, employment status, English proficiency) and racism experience did not predict COVID-19 vaccine accessibility (Table 3). 

When predicting COVID-19 vaccine safety concerns, South Asians (compared to Non-Asians) reported significantly higher safety concerns regarding COVID-19 vaccines. However, no significant difference was found between East or Southeast Asians compared to Non-Asians. In addition, lower English proficiency and greater racism experience were predictive of greater COVID-19 vaccine safety concerns. None of the other demographic variables was found to significantly predict COVID-19 vaccine safety concerns.

Finally, regarding trust in public health agencies in terms of COVID-19 vaccine recommendations, demographic characteristics as well as racism experience were not predictive. However, those who received some college education had less trust in public health agencies compared to those with less than a high school diploma education level. 

Next, using the aforementioned results which showed that racism was predictive of COVID-19 vaccine safety concerns, we further examined whether racism experience mediates the association between race/ethnicity and COVID-19 vaccine safety concerns. The SEM analyses found that racism experience significantly mediated the association between race/ethnicity and COVID-19 vaccine safety concerns. Specifically, compared to Non-Asians, (a) East Asians reported higher racism experience, which, in turn, predicted more COVID-19 vaccine safety concerns (*B_indirect_* = 0.06, *SE* = 0.03, *p* = 0.03); (b) South Asians reported higher racism experience, which, in turn, predicted more COVID-19 vaccine safety concerns (*B_indirect_* = 0.04, *SE* = 0.02, *p* = 0.04); and (c) Southeast Asians reported higher racism experience, which, in turn, predicted more COVID-19 vaccine safety concerns (*B_indirect_* = 0.05, *SE* = 0.02, *p* = 0.04).

### 3.4. Social Vulnerability Index and COVID-19 Concerns

**Figure 2:**
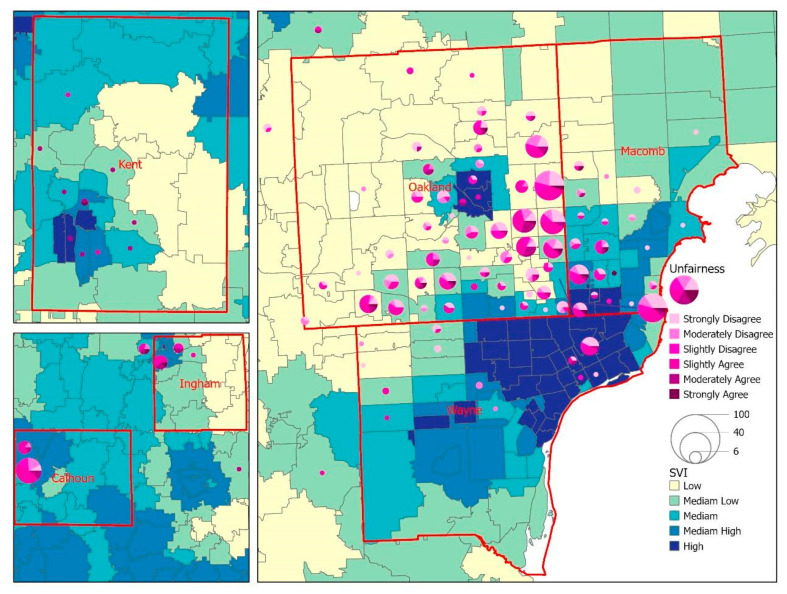
A map for the visualization of the overall SVI against the perception of unfairness due to race/ethnicity among survey respondents.

**Figure 3:**
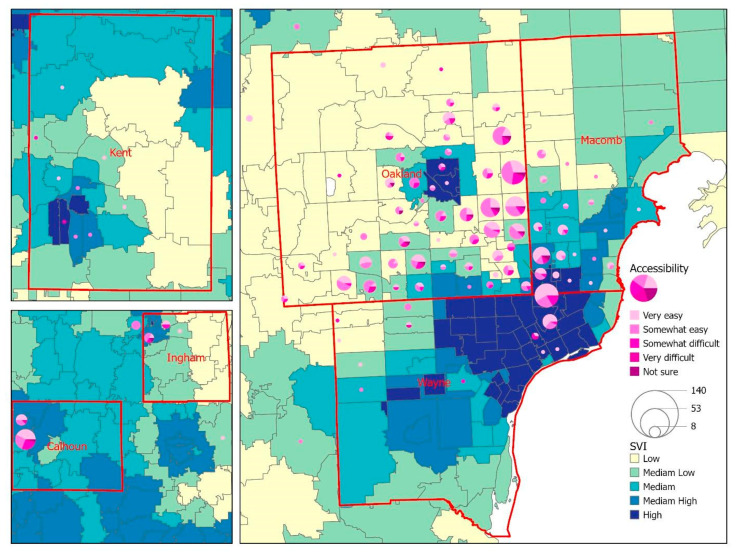
A map for the visualization of the overall SVI against the accessibility of COVID-19 vaccination among survey respondents.

**Figure 4:**
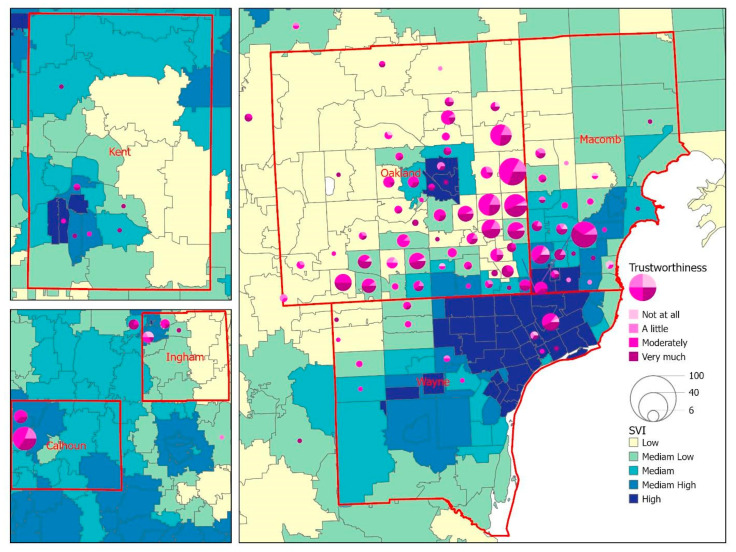
A map for the visualization of the overall SVI against the trust in public health agencies among survey respondents.

The geographic distribution of the survey respondents revealed that the majority of respondents (86.8%) resided in the tri-county region (Wayne, Oakland, and Macomb) of southeast Michigan. Respondents from Calhoun, Ingham, and Kent counties represented central and west Michigan (Figure 2). Figure 2 displays a choropleth map to visualize the overall SVI by zip code area, with the lighter color indicating a lower level of SVI and the darker color showing a zip code area with higher vulnerability. The overlay analysis between SVI and unfairness in Figure 2 shows that respondents living in low-to-moderate SVI areas reported unfairness because of race/ethnicity. Regarding access to COVID-19 vaccines (Figure 3), the geographic patterns between SVI and vaccine accessibility tended to be generalized across the six counties, while we did notice a slightly higher number of difficult-to-very difficult response rates in zip codes with a high SVI. A similar pattern was noted when exploring the relationship between SVI and trust in public health agencies (Figure 4), with respondents from a few zip code areas with high SVI having less trust.

## 4. Discussion

Racial and ethnic minorities are disproportionately impacted by the COVID-19 pandemic in the U.S. While Asian American vaccine acceptance has been found to be higher compared to other racial/ethnic groups [35], our study sought to provide new insights into COVID-19 vaccination uptake by investigating the mechanisms behind vaccine hesitancy and how social vulnerability can affect perceptions of COVID-19 vaccine safety, accessibility, and trust in public health agencies. In addition, from a health equity perspective, our study provided disaggregated data for three Asian American subgroups, such data being scarce in the literature. 

This study found that East Asians (compared to Non-Asians) had significantly more challenges when it came to accessing COVID-19 vaccines, while South Asians (compared to Non-Asians) reported significantly higher safety concerns regarding COVID-19 vaccines. Study findings can inform public health officials, community partners, and healthcare system outreach programs to improve vaccine acceptance and, as a result, provide better protection against COVID-19, which has disproportionately affected these Asian American groups [36]. 

Research on COVID-19 vaccine acceptability is in its infancy. Therefore, it is important to identify factors that may predict hesitation about COVID-19 vaccines with the goal of increasing the acceptability of and the confidence of the population in COVID-19 vaccines. The current study set out to examine the extent to which the intersection of racism and demographics impacts concerns about contracting COVID-19, vaccine safety, accessibility, and trust in public health agencies with respect to COVID-19. We found that racism experience mediates the associations between safety concerns about COVID-19 vaccines and race/ethnicity. Specifically, three Asian subgroups (East Asians, South Asians, and Southeast Asians) experienced more racism (compared to Non-Asians) and greater safety concerns. In addition, East Asians reported more challenges in accessing COVID-19 vaccines and experienced higher levels of racism, which, in combination, made vaccine uptake more challenging. Targeted efforts can provide support for this group to address negative perceptions and increase COVID-19 vaccine accessibility to improve vaccine uptake. These findings are among the first to demonstrate the complex mechanisms connecting demographics, racism, and perceptions related to COVID-19 vaccine confidence. 

One of the principal factors associated with attitude towards and perception of COVID-19 vaccination is English proficiency. Our study findings showed that people with limited English proficiency were more concerned about getting COVID-19 and vaccine safety. In addition to reporting experience of more unfair treatment and safety concerns due to race, Asian American respondents with limited English proficiency may face greater challenges in accessing accurate and timely information about the course of the pandemic, including updates about vaccines and effective mitigation measures. More support is needed to disseminate language-specific and culturally appropriate messages at suitable community venues (e.g., faith-based organizations, community centers, and cultural grocery stores) and deploy bilingual trusted messengers, who are crucial to increasing vaccine acceptance and ultimately vaccine uptake.

During the pandemic, socially vulnerable populations experienced disproportionate adverse outcomes as a result of social determinants of health (SDOH) factors, for example, healthcare access and quality, employment, education, and racism and discrimination [37]. Our study has shown unique geographic patterns between SVI areas and racism-related perceptions, and the findings have revealed that respondents living in lower SVI areas had fewer unfairness perceptions while those living in higher SVI areas had lower vaccine accessibility and trust in public health agencies. Additional resources can be allocated to socially vulnerable areas to mitigate the negative effects on health stemming from SDOH factors and racial discrimination and unfair treatment [26].

### Limitations

There are several limitations to this study. Our cross-sectional study design may limit the temporal causality that can be inferred between the latent variables and the outcomes of interest in the study. Furthermore, due to the nature of convenience sampling, the generalizability of our findings may be limited. In addition, given that the respondents completed the surveys voluntarily, the respondents might have had greater concerns and perceptions regarding COVID-19 and COVID-19 vaccine-related issues, raising the question of self-selection bias. Future studies may consider using a probability-based sample to enhance the generalizability of study findings. Finally, because the numbers of some Asian groups (e.g., Asian Indians) and the Non-Asian group in our study were small, future research is encouraged to consider increasing outreach efforts to recruit more respondents from these groups; as a result, researchers will be able to present disaggregated data for Asian Americans from each racial and ethnic group.

## 5. Conclusions

The current study strengthens the literature and advances our understanding of racism, social vulnerability, and COVID-19 vaccine-related attitudes among Asian Americans. It is important to elucidate these issues because negative attitudes toward vaccination reduce vaccine uptake, which is a public health concern [38]. These insights remind us of the dynamics of individuals’ attitudes, bringing us closer to understanding the underlying mechanisms that influence attitudes and behaviors. Our findings suggest that policy makers and community leaders should tailor vaccine information and efforts to those with limited English proficiency and Asian American groups that experience greater challenges in terms of accessibility and vaccine safety concerns and address knowledge concerns that are prevalent and likely modifiable. A multi-pronged approach with culturally appropriate and tailored health communication and education outreach is critical to achieving the goal of health equity for Asian Americans and improving public health in the U.S.

## Figures and Tables

**Table 1:** Descriptive statistics for study variables (total *N* = 617).

**Categorical Variables**	**Available *N***	**%**
Gender		
Male	231	41.18
Female	330	58.82
Ethnicity		
East Asian	260	42.76
South Asian	100	16.45
Southeast Asian	179	29.44
Non-Asian	69	11.35
Education		
High school or less	44	20.87
High school graduate	110	18.36
Some college	125	20.87
Bachelor’s or higher	320	53.42
Employment status		
Employed	414	69.70
Unemployed	180	30.30
**Continuous Variables**	**Available *N***	***M (SD)***
Age	570	40.91 (14.23)
English proficiency	607	3.23 (0.83)
Accessibility of COVID-19 vaccination		
Number of difficulties	601	0.80 (0.63)
General perceived difficulty	588	1.80 (0.80)
General concerns about COVID-19	589	2.32 (1.01)
COVID-19 vaccine concerns		
Number of concerns	593	0.90 (0.79)
General safety concerns	592	3.15 (0.74)
Trust in public health agencies	573	3.21 (0.76)
Racism		
Being treated unfairly	585	2.92 (1.53)
Safety concern due to one’s race	586	3.16 (1.78)

**Table 2:** Means and standard deviations for the race/ethnicity groups related to their racism experiences.

	Non-Asian	East Asian	South Asian	Southeast Asian	*F*	*MS_error_*	*p*	*η²*
Being treated unfairly	1.54 (1.18) ^b^	3.21 (1.44) ^a^	2.97 (1.63) ^a^	3.00 (1.45) ^a^	24.21	2.08	<0.001	0.11
Safety concern due to one’s race	1.26 (0.73) ^c^	3.74 (1.61) ^a^	2.91 (1.93) ^b^	3.21 (1.72) ^b^	42.46	2.62	<0.001	0.18

Notes: For each racism variable, group means with a different superscript are significantly different at *p* < 0.05 according to post-hoc Tukey tests.

**Table 3:** Regression coefficients predicting COVID-19 concerns, vaccine accessibility, vaccine concerns, and trust in public health agencies.

	COVID-19 Concerns		Vaccine Accessibility		Vaccine Safety Concerns		Trustworthiness	
	*B (SE)*	*p*	*B (SE)*	*p*	*B (SE)*	*p*	*B (SE)*	*p*
Sex at birth (ref.: female)								
Male	−0.332 (0.087)	<0.001	−0.036 (0.063)	0.567	−0.043 (0.047)	0.363	−0.013 (0.067)	0.849
Age *	−0.012 (0.003)	<0.001	−0.004 (0.002)	0.065	−0.001 (0.002)	0.545	0.004 (0.003)	0.145
Ethnicity (ref.: Non-Asian)								
East Asian	−0.351 (0.171)	0.040	0.320 (0.121)	0.008	0.176 (0.093)	0.059	−0.001 (0.133)	0.994
South Asian	−0.036 (0.181)	0.842	0.161 (0.124)	0.194	0.226 (0.097)	0.020	0.172 (0.139)	0.218
Southeast Asian	0.038 (0.164)	0.817	0.115 (0.112)	0.304	0.128 (0.088)	0.145	−0.004 (0.126)	0.974
Education (ref: <high school)								
High school or equivalent (GED)	−0.399 (0.189)	0.035	−0.068 (0.128)	0.594	0.092 (0.102)	0.368	−0.114 (0.154)	0.459
Some college	−0.082 (0.191)	0.667	0.037 (0.131)	0.776	0.194 (0.104)	0.062	−0.345 (0.155)	0.026
Bachelor’s degree or higher	0.010 (0.182)	0.956	0.179 (0.132)	0.174	0.164 (0.099)	0.098	−0.169 (0.148)	0.255
Employment status (ref.: unemployed)								
Employed	−0.071 (0.097)	0.464	−0.069 (0.067)	0.306	−0.035 (0.052)	0.503	0.118 (0.074)	0.111
English proficiency	0.119 (0.060)	0.046	−0.053 (0.041)	0.195	−0.076 (0.032)	0.017	0.028 (0.046)	0.546
Racism experience	0.092 (0.060)	0.127	0.042 (0.042)	0.313	0.067 (0.032)	0.038	−0.069 (0.047)	0.147

Notes: *B* = unstandardized regression weights, *SE* = standard error. * Age was entered as a continuous variable.

## Data Availability

Access to the dataset is not possible due to ethical approval restrictions.
